# Pulmonary Hypertension: Diagnosis and Management

**DOI:** 10.3390/diagnostics11061066

**Published:** 2021-06-09

**Authors:** Adam Torbicki

**Affiliations:** Department of Pulmonary Circulation, Thromboembolic Diseases and Cardiology, Center for Postgraduate Medical Education, ECZ-Otwock, 05-400 Otwock, Poland; adam.torbicki@ecz-otwock.pl

With great satisfaction, we now share with you the contents of this Special Issue of *Diagnostics* dedicated to Diagnosis and Treatment of Pulmonary Hypertension (PH). Our satisfaction is due to the outstanding contributions from leading PH expert centers worldwide. Those contributions include original data as well as excellent reviews of the state of the art and future perspectives covering several critically important problems related to PH.

As an introduction, we invite you to a journey across the ages to discover milestones in understanding human pulmonary circulation. I have the pleasure to bring you from ancient China and Egypt through the Middle Ages, Renaissance and modern history, up to the moment when the first successful implementation of a human-made heart–lung machine definitively proved that our concepts of pulmonary circulation finally became correct [1].

The first section contains five papers dedicated to problems related to overlap of vascular and respiratory problems in the lungs—Olschewski’s perspective from Ludwig Boltzmann Institute, University of Graz [2] is followed by a review of the state of the art in PAH associated with sclerodermia by Naranjo and Hassoun, from John Hopkins University, Baltimore [3]. The modern approach to thoracic imaging in patients with PH, including machine learning and artificial intelligence, is covered by outstanding reviews from a team from Sheffield University [4], and Gibbs and Gopalan from Imperial College, London and Cambridge University, respectively [5], while an attempt to link genetic background with radiological phenotyping in PVOD is reported from Hospital Universitario Doce de Octubre in Madrid [6].

From differential diagnosis, we move to risk stratification in PH with new biomarkers suggested by a group from Rome [7], and a multiparametric non-invasive approach from Otwock, Poland [8]. An important, unexplored possibility of using an echocardiographically assessed right ventricular reserve as a prognostic factor is suggested from Heidelberg [9]. This part is completed by a comprehensive overview of the role of echocardiography for the assessment of PH in congenital heart disease in the young prepared by an expert group of pediatric cardiologists from Austria, Germany and the UK [10].

The impact of the COVID-19 pandemic on pulmonary hypertension is still unclear. This Special Issue presents two perspectives on its significance—an original report from a large University Hospital in New York City [11] and a review from Spain [12], which was particularly heavily hit by the first wave of the pandemic, in 2020.

Finally, two interventional methods still searching their optimal place within therapeutic strategy in PH are covered by five papers. An interesting report from the Bakoulev National Medical Research Center of Cardiovascular Surgery in Moscow on a large series of stent-protected atrial septostomies [13] is commented on by Prof Sandoval [14], a global expert in this method from the Instituto Nacional de Cardiologia in Mexico City. Three original contributions deal with chronic thromboembolic pulmonary hypertension (CTEPH). A paper from Madrid addresses the issue of its operability [15], crucial for the qualification to balloon pulmonary angioplasty (BPA), while Banaszkiewicz et al. from Otwock reports on a new marker associated with early complications of BPA [16]. Finally, a case report from our center, by Darocha et al., points at the possible role of BPA with stenting in cases of CTEPH with proximal stenoses prone to elastic recoil when surgical treatment is impossible due to severe comorbidities or decision of the patient [17].

Please pick your choices but also think about sharing your own data and experience by publishing on the open access pages of *Diagnostics*. You will be supported by an editorial team which—as the Guest Editor of this Special Issue—I found very friendly and efficient.
